# Gentiopicroside Attenuates Lithium/Pilocarpine-Induced Epilepsy Seizures by Down-Regulating NR2B/CaMKII/CREB and TLR4/NF-κB Signaling Pathways in the Hippocampus of Mice

**DOI:** 10.3390/ph17111413

**Published:** 2024-10-22

**Authors:** Miao-Miao Tian, Gang Liu, Juan Du, Yue Liu, Wei Wei, Xiao-Bing Lan, Dong-Mei Hai, Lin Ma, Jian-Qiang Yu, Ning Liu

**Affiliations:** 1 School of Pharmacy, Ningxia Medical University, Yinchuan 750004, China19870041@nxmu.edu.cn (J.-Q.Y.); 2 Key Laboratory of Protection, Development and Utilization of Medicinal Resources in Liupanshan Area, Ministry of Education, Yinchuan 750004, China; 3 Colaborative Innovation Center for Ningxia Characteristic Traditional Chinese Medicine by Ningxia Hui Autonomous Region & Education Ministry of P.R. China, Yinchuan 750004, China; 4 New Drug Screening Center, State Key Laboratory of Natural Medicines, China Pharmaceutical University, Nanjing 210009, China

**Keywords:** epilepsy, gentiopicroside, pilocarpine, NR2B/CaMKII/CREB signaling pathway, TLR4/NF-κB signaling pathway, apoptosis, neuroinflammatory

## Abstract

**Background:** Epilepsy is a prevalent and disabling neurological condition characterized by recurrent seizures. Approximately 50% of adults with active epilepsy have at least one comorbidity and they are at a greater risk of premature death than the general population. Gentiopicroside (Gent) is a primary component of *Gentiana macrophylla* Pall. that has been shown to have diverse pharmacological properties. However, its role in epileptic seizures in adult mice and its underlying mechanism of action remain obscure. We aimed to explore the anti-epileptic effect and mechanism of Gent on lithium/pilocarpine (Pilo)-induced epilepsy seizures in mice. **Methods:** In this study, we established a lithium/Pilo-induced epilepsy model, and Gent was first given to mice 30 min before Pilo administration. Then, we detected behavioral and histopathological changes through electrocorticographic (ECoG) measurements, Nissl staining, Fluoro-Jade B (FJB) staining, and immunohistochemical staining. We then used molecular biology techniques, such as Western blotting, quantitative polymerase chain reaction (qPCR) analysis, and the enzyme-linked immunosorbent assay (ELISA) to investigate the mechanisms of Gent in lithium/Pilo-induced epileptic seizures in mice and lipopolysaccharide (LPS)-induced inflammatory astrocytes. **Results:** We confirmed that Gent could prevent abnormal ECoG activity, behavioral changes, and neurodegeneration. Subsequently, we found Gent could downregulate the factors that could promote apoptosis (i.e., the NR2B/CaMKII/CREB signaling cascade) and neuroinflammatory-related factors (i.e., the TLR4/NF-κB signaling cascade). **Conclusions:** Gent could be a potential therapeutic agent for epilepsy, offering possibilities for both prevention and treatment. Our research establishes a preliminary experimental framework for ongoing studies into Gent’s efficacy as a treatment for epilepsy.

## 1. Introduction

Epilepsy is a chronic neurological disease caused by the aberrant synchronized firing of populations of neurons primarily due to an imbalance between excitatory and inhibitory neurotransmission [1]. It is characterized by an enduring propensity for seizures, which can lead to neural damage and the development of associated dysfunctions such as cognitive, locomotor, and sensory disturbances [2]. This neurological disorder affects around 65 million people worldwide and an estimated 10 million people in China alone. The average daily cost of inpatient care for patients with epilepsy is USD 150, while status epilepticus costs USD 225, which places an enormous burden on patients’ quality of life [1,3,4,5]. Presently, pharmacotherapy is the first choice for epilepsy treatment. Over 20 antiseizure drugs are available for treating epileptic seizures, but nearly one in three epileptic patients have seizures that are unresponsive to pharmaceuticals. Therefore, patients suffering from drug-resistant epilepsy have higher clinical risks of perceived injuries, psychological disorders, and premature death [6]. Thus, there is an urgent need to study the pathophysiological processes of epilepsy and develop mechanism-oriented antiepileptic drugs.

It is believed that excessive N-methyl-D-aspartate (NMDA) receptor activation contributes to status epilepticus pathophysiology [7]. The academic community widely recognizes that an imbalance in gamma-aminobutyric acid (GABA) and glutamate neurotransmission is a pivotal factor in the etiology of epilepsy, as evidenced in a substantial body of experimental research [8]. Seizures are linked to an amplification of glutamate responses. The NMDA receptor-mediated process initiates a signaling cascade that underpins a series of subsequent biological activities, including excitotoxicity, excessive calcium influx, and cellular death [9,10]. Moreover, extensive research suggests that the NMDA receptor subtype 2B (NR2B) is integral to the etiology and progression of epileptic conditions [11,12].

In the 1980s, Turski and Cavalheiro introduced the experimental pharmacological model of epilepsy secondary to status epilepticus (SE) induced by the administration of pilocarpine (Pilo). This model was later modified by the prior administration of lithium salts, becoming the lithium/Pilo experimental model widely used in the literature, which best represents temporal lobe epilepsies (TLEs), the most common drug-resistant epilepsy in adult humans [13]. Additionally, TLE is directly related to morphological changes such as mesial temporal sclerosis, gliosis, and inflammation [14]. Therefore, in this study, we use the lithium/Pilo-induced epileptic seizures model to study the mechanisms and potential treatments of epilepsy. This model can be used to determine epileptogenic mechanisms that might upregulate NR2B subunit expression in both primary hippocampal cultures and the hippocampus of epilepsy-seizure rats [15,16,17]. During an epileptic episode, increased NMDA receptor expression leads to prolonged calcium channel opening, causing excessive intracellular calcium accumulation. This results in mitochondrial depolarization and overactivation of calcium-dependent signaling pathways, which could lead to apoptosis [18]. The enzyme calcium/calmodulin-dependent protein kinase II (CaMKII) is highly adaptable and crucial for facilitating numerous calcium-regulated processes within neurons [19]. The activation of CaMKII depends on calcium activation. Dysregulation of CaMKII activity has been documented in a range of neurological disorders characterized by disrupted calcium homeostasis, including stroke, seizures, and traumatic brain injury [20,21]. The cyclic adenosine monophosphate (cAMP)-response element-binding protein (CREB) acts as a key regulatory factor in the genesis of epilepsy, involving its onset and progression. CREB directly regulates transcription through phosphorylation. CaMKII activates CREB by phosphorylating it, thereby boosting its transcriptional activity. Notably, the immunostaining intensity of phosphorylated CREB at serine 133 (Ser133), an indicator of CREB activation, is significantly elevated in the hippocampus of mice subjected to lithium/Pilo-induced convulsions compared to control mice [22,23]. Moreover, experimental models of epilepsy have demonstrated that seizures trigger concurrent necrotic and apoptotic cell death [24]. Under epileptic conditions, apoptosis-related proteins undergo changes and there is a downregulation of mRNA and protein expression of the anti-apoptotic protein B-cell leukemia/lymphoma 2 (Bcl-2) in mice, which is concomitant with an upregulation of mRNA and protein expression of the pro-apoptotic proteins Bcl-2-associated X protein (Bax) and caspase-3, but reversing these alterations can mitigate neuronal damage and apoptosis in epileptic mice, thereby conferring a neuroprotective effect and ameliorating epilepsy symptoms [8,25].

Meanwhile, existing research indicates that the inflammatory response is indispensable in both the initiation and progression of epilepsy. Astrocytes, which are the predominant type of glial cells in the central nervous system, have a crucial function in regulating the brain’s inflammatory reactions and are intimately associated with epilepsy. Upon epileptic injury, astrocytes and other glial cells undergo rapid activation, characterized by both morphological and functional alterations. This activation results in the release of diverse pro-inflammatory cytokines, such as interleukin-1 beta (IL-1β), tumor necrosis factor-α (TNF-α), and interleukin-6 (IL-6). These inflammatory agents are capable of triggering Toll-like receptors (TLRs), thereby initiating immune cell responses. Meanwhile, they are implicated in the pathogenesis of epilepsy. TLR4, the first identified subtype of TLRs, can be activated by these inflammatory mediators and then promote excitatory synaptic development. This process increases neuronal excitability and susceptibility to epilepsy, ultimately facilitating epileptic seizures. Inhibition of TLR4 expression has been shown to effectively mitigate epileptic seizures [26,27,28,29]. Nuclear factor kappa-B (NF-κB) serves as an essential downstream element within the TLR4 signaling cascade, crucial for regulating inflammation. Under normal physiological conditions, NF-κB resides in the cytoplasm as part of inactive complexes, bound to the inhibitory protein inhibitor of nuclear factor kaineppa-B alpha (IκB-α). Upon activation by IκB kinase (IKK), IκB-α undergoes phosphorylation, leading to its ubiquitination and subsequent degradation, which in turn activates NF-κB. In temporal lobe epilepsy patient tissues, the activation of NF-κB has been detected. Once activated, NF-κB’s translocation to the nucleus promotes the production of inflammatory cytokines such as IL-1β, TNF-α, and IL-6; these mediators can initiate further inflammatory cascades [30]. After brain injury, it can cause neuroinflammatory reactions characterized by an increase in pro-inflammatory cytokines, which mediate recurrent epileptic seizures [31,32]. Pro-inflammatory cytokines such as IL-1β, IL-6, and TNF-α typically have low concentrations in the brain and increase after epileptic seizures; these key cytokines are involved in various mechanistic inflammatory pathways that can lead to harmful synaptic changes and neuronal excitability. Therefore, inhibiting pro-inflammatory cytokines may play a key role in the treatment of epilepsy [33,34].

**Figure 1:**
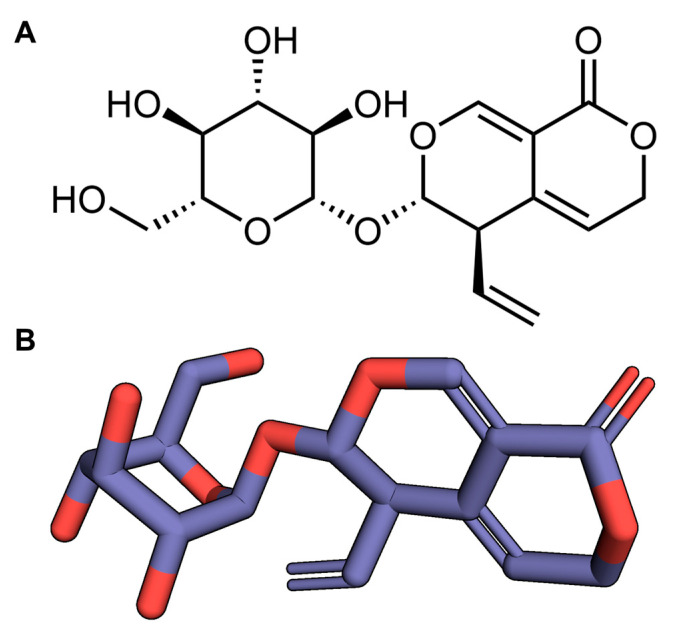
Structure of gentiopicroside. (**A**) Molecular formula. (**B**) 3D structure. The highlighted red part represents oxygen atoms.

Gentiopicroside (Gent) is a secoiridoid glycoside with the aglycone being iridoid alcohol. The bond between C_7_–C_8_ in the cyclopentane ring of the aglycone is broken, and after ring cleavage, C_7_ forms a six-membered lactone ring with C_11_; its hemiacetal C_1_–OH is unstable, so it combines with glucose to form Gent. The molecular formula for Gent is C_16_H_20_O_9_, and its molecular weight is 356.33 (Figure 1). Gent is the predominant bioactive compound extracted from *Gentiana macrophylla* Pall. and exhibits a broad spectrum of pharmacological properties, such as anti-apoptotic, antioxidant, anti-inflammatory, and analgesic effects [35,36,37]. More specifically, the analgesic effects of Gent in persistent inflammatory pain is linked to the downregulation of NR2B receptors. The anti-inflammatory activity of Gent in non-alcoholic steatohepatitis occurs by suppressing the TLR4/NLRP3 signaling pathway [37,38]. Our previous study found that Gent mitigated epileptogenesis in immature rats by inactivating the (NOD-like receptor protein 3) NLRP3 inflammasome through the inhibition of P2X7 receptor expression [39]. However, whether Gent ameliorates epileptic seizures triggered by lithium/Pilo in adult rats remains largely unknown. Therefore, the aim of this study is to determine the anti-epileptogenic efficacy of Gent in adult mice induced by lithium/Pilo, with a particular emphasis on its effects on the NR2B/CaMKII/CREB and TLR4/NF-κB signaling pathways.

## 2. Results

### 2.1. Gent Normalized ECoG Measurements in Lithium/Pilo-Induced Seizures

ECoG is a widely used diagnostic instrument for conditions including epilepsy, stroke, and traumatic brain injury. It functions by recording the spontaneous electrical activity of neuronal assemblies via electrodes. Early indicators of epilepsy following epileptic seizures include abnormal brain function, a marked increase in neuronal excitability, an elevation in total cortical power, and alterations in ECoG patterns.

**Figure 2:**
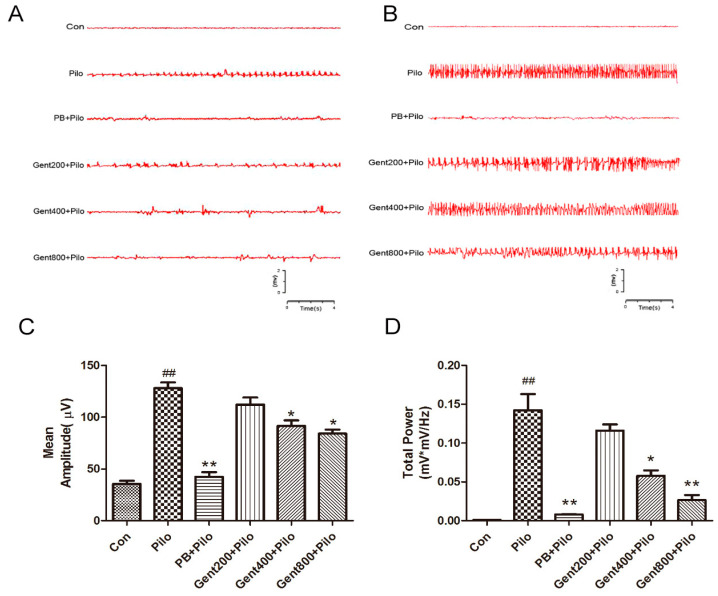
Electrocorticography recordings from adult mice after lithium/Pilo administration. (**A**) Exemplary waveforms captured 10 min after pilocarpine injection are displayed. (**B**) Representative samples recorded maximum seizure in 30 min. (**C**) Mean amplitude of seizure ECoGs was quantified for comparison. (**D**) Total power was quantified for comparison. Results are expressed as mean ± SEM (n = 8). ^##^ *p* < 0.01 versus control group; * *p* < 0.05, ** *p* < 0.01 versus Pilo group.

Representative results for ECoG are shown in Figure 2. The ECoG recordings obtained from the lithium/Pilo-treated mice displayed unusual rhythms, including spikes with high amplitudes, numerous occurrences of polyspikes, spike–wave complexes, and an increased average amplitude of spikes and total power (*p* < 0.01). Pretreatment with Gent at dosages of 400 and 800 mg/kg effectively mitigated aberrations in ECoG recordings by improving abnormal cortical brain rhythms and decreasing the average amplitude of spikes and total power (*p* < 0.05, *p* < 0.01, respectively). These data verify the presence of seizure after lithium/Pilo treatment and suggest that Gent restores lithium/Pilo-induced abnormal ECoG activity in the brain.

### 2.2. Gent Improved Behavioral Measurements in Lithium/Pilo-Induced Seizures

High-grade seizures (classes IV–V), generalized tonic convulsion, and clonic convulsion in all four limbs, along with recumbent, rearing, and lethal outcomes, were noted in the entire cohort of mice, with the exception of those in the control (Con) and phenobarbital (PB) + Pilo groups. Class I–III low-grade seizure behaviors, including head nodding and involuntary rhythmic contractions of the muscles in the forelimbs, were displayed by the PB + Pilo group of mice.

**Figure 3:**
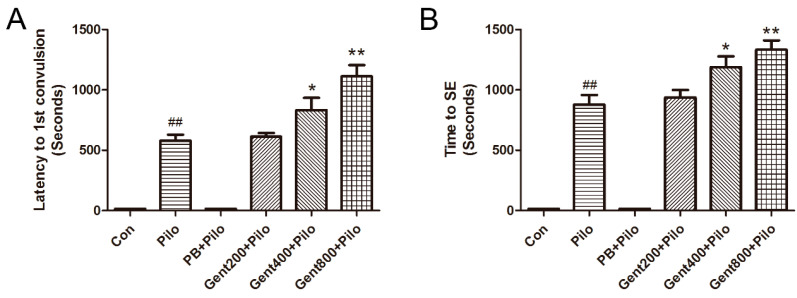
Effects of gentiopicroside on lithium/Pilo-induced behavioral changes in adult male mice (n = 16 per group). (**A**) Latency to first convulsion. (**B**) Time to SE. Results are expressed as mean ± SEM. ^##^ *p* < 0.01 versus Con group; * *p* < 0.05, ** *p* < 0.01 versus Pilo group.

No instances of seizures were detected in any of the mice within the Con group. Class I-III low-grade seizure behaviors, including head nodding and involuntary rhythmic contractions of the muscles in the forelimbs, were displayed by the PB + Pilo group mice. In contrast to the Pilo group, the latency to the first convulsion in the Gent groups (400 and 800 mg/kg) was noticeably longer. Nevertheless, the administration of Gent (200 mg/kg) did not yield a statistically significant effect on the latency to the first occurrence of convulsion when compared to the Pilo group (*p* > 0.05). High Gent dosages administered beforehand resulted in a considerable prolongation of the time to SE in mice, specifically at 400 and 800 mg/kg (*p* < 0.05, *p* < 0.01, respectively), as shown in Figure 3. Consequently, there was a notable decrease in the incidence of SE (75% and 56.2%, respectively).

In addition, it was observed that 62.5% (10 out of 16) of the mice in the Pilo group survived, while all mice (16 out of 16) in the high-Gent dosage groups (400 and 800 mg/kg) survived. It is evident from Table 1 that all mice in the Con, PB + Pilo, Gent400 + Pilo, and Gent800 + Pilo groups survived. The data show that pretreatment with Gent effectively alleviated the epileptic convulsions generated by lithium/Pilo and resulted in a decrease in the mortality rate amongst the mice.

### 2.3. Gent Pretreatment Decreased Lithium/Pilo-Induced Neurodegeneration

**Figure 4:**
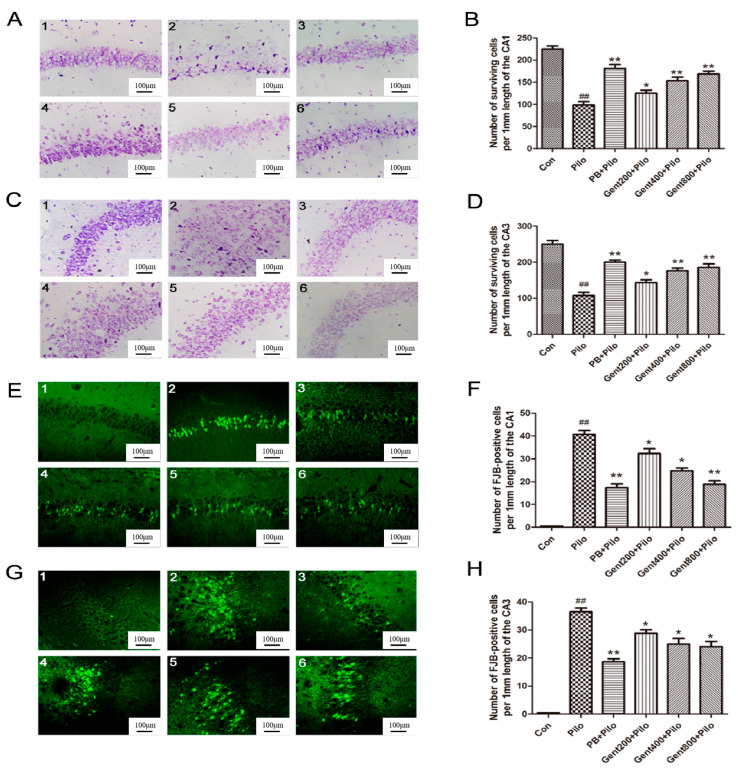
Effects of gentiopicroside on lithium/Pilo-induced neurodegeneration. (**A**) Nissl staining of the pyramidal neurons in the CA1 region of the hippocampus. (**C**) Nissl staining of the pyramidal neurons in the CA3 region of the hippocampus. (**E**) FJB staining of hippocampal CA1 pyramidal neurons. (**G**) FJB staining of hippocampal CA3 pyramidal neuronal. (**1**) Con group. (**2**) Pilo group. (**3**) PB + Pilo group. (**4**) Gent200 + Pilo group. (**5**) Gent400 + Pilo group. (**6**) Gent800 + Pilo group. Scale bar = 100 μm. Quantitative analysis of surviving neurons and FJB-positive cells in CA1 (**B**,**F**) and CA3 (**D**,**H**) regions (n = 4 per group, each animal was examined in six sections). Results are expressed as mean ± SEM. ^##^ *p* < 0.01 versus the control group; * *p* < 0.05, ** *p* < 0.01 versus the Pilo group.

Nissl staining serves as a histopathological technique for assessing alterations in Nissl bodies within the neuronal cytoplasm and identifying neuronal damage. The Con group exhibited a high count of hippocampal neurons that were well-organized and abundant in Nissl Bodies. In the Pilo group, the hippocampal region demonstrated neuronal death, tissue degradation, vacuolization, fewer Nissl bodies, and a significant reduction in neuronal density; and displayed a significant reduction in the quantity of surviving neurons (*p* < 0.01). Pretreatment with Gent significantly mitigated vacuolization and neuronal loss, thereby restoring normal cellular morphology. Compared with the Pilo group, administration of Gent (400 and 800 mg/kg) led to an enhancement in neuronal survival (*p* < 0.05, *p* < 0.01, respectively), as shown in Figure 4.

Fluoro-Jade B (FJB) staining serves as a histopathological technique for detecting neuronal degeneration and necrosis. Seventy-two hours post-initiation of convulsions or SE, a limited number of cells in the Con group exhibited positive FJB staining. In comparison, a significant loss of neurons was identified within the hippocampal CA1 and CA3 regions in lithium/Pilo-treated mice (*p* < 0.01). Administration of Gent 30 min before the lithium/Pilo treatment markedly suppressed the increase in FJB-positive cells induced by convulsions or SE (*p* < 0.05, *p* < 0.01, respectively) (Figure 4).

In summary, Gent administration exhibited a neuroprotective effect in lithium/Pilo-induced epilepsy via attenuating neuronal cell death, diminishing the depletion of Nissl bodies, and alleviating brain damage.

### 2.4. Gent Inhibited Lithium/Pilo-Induced Activation and Proliferation of Astrocytes

The glial fibrillary acidic protein (GFAP) is an astrocytic cytoskeletal protein that serves as a sensitive and reliable marker of abnormal activation and proliferation of astrocytes due to neuronal damage [40]. Therefore, we used it as a target index in our study.

**Figure 5:**
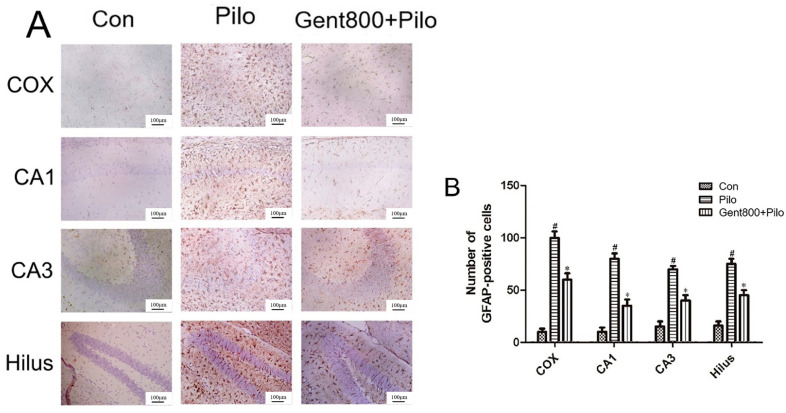
Effects of gentiopicroside on lithium/Pilo-induced activation and proliferation of astrocytes. (**A**) Immunohistochemical staining. Scale bar = 100 μm. (**B**) Quantitative analysis of GFAP-positive cells. Data are represented as the mean ± SEM from six independent experiments (n = 6 per group, each animal was examined in six sections). ^#^ *p* < 0.05 versus the control group; * *p* < 0.05 versus the Pilo group.

As illustrated in Figure 5, marked disparities in the morphology of astrocytes were noted after administrating lithium/Pilo to mice. Specifically, the Con group exhibited fewer slender, star-shaped astrocytes in the cerebral cortex, hippocampal CA1 and CA3 regions, and hilus regions. In contrast, the Pilo group demonstrated a significant increase in activated astrocytes characterized by elongated cell body hypertrophy (*p* < 0.05). Gent treatment significantly inhibited astrocyte activation in the cerebral cortex, hippocampal CA1 and CA3 regions, and hilus regions, and significantly lowered the expression of GFAP-positive cells (*p* < 0.05). These results indicate that Gent inhibits the activation and proliferation of astrocytes triggered by epileptic seizures.

### 2.5. Gent Regulating Apoptosis-Related Factors

#### 2.5.1. Gent Diminished the NR2B/CaMKII/CREB Signaling Pathway in the Hippocampus of Lithium/Pilo-Induced Epilepsy-Seizure Mice

Total protein encompasses both phosphorylated and non-phosphorylated proteins. The enzyme that catalyzes phosphorylation modification is kinase. Phosphorylation represents an activated state of the protein, resulting in alterations to its original conformation. This conformational change subsequently activates the protein’s activity, thereby influencing downstream signaling pathways. CaMKII and CREB are total proteins, while p-CaMKII and p-CREB are phosphorylated proteins.

The activation and function of CaMKII are regulated through its phosphorylation state. The self-phosphorylation of threonine 286 (Thr286) is the most critical, as it increases the affinity of CaMKII for calcium ions and enables CaMKII to maintain activity even after a decrease in calcium ion levels, known as autonomous activity [41].

As an important nuclear regulatory factor, the activity status of CREB mainly depends on whether phosphorylation occurs, especially at the serine 133 (Ser133) site. p-CREB can more effectively bind to specific DNA sequences, such as cAMP response elements (CREs), thereby activating transcription of downstream genes [42].

**Figure 6:**
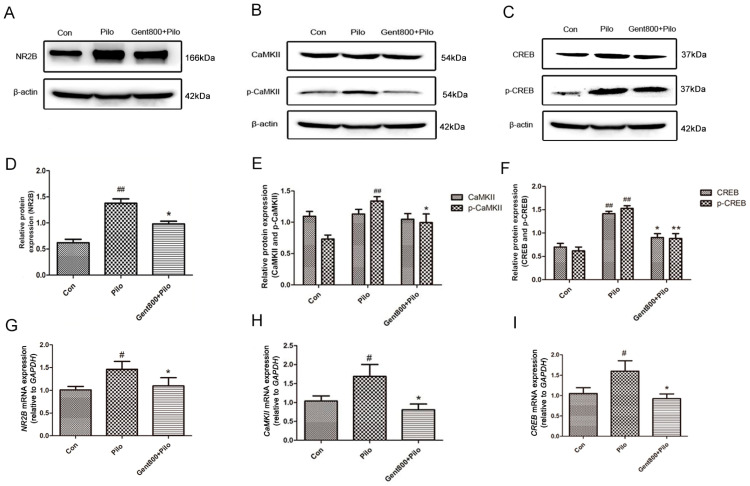
Gentiopicroside diminished the NR2B/CaMKII/CREB signaling pathway (n = 6 per group). (**A**–**C**) Western blot analysis of NR2B, CaMKII, p-CaMKII, CREB, and p-CREB. (**D**–**F**) Quantitative analysis of NR2B, CaMKII, p-CaMKII, CREB, and p-CREB. (**G**–**I**) Relative quantitative mRNA levels of NR2B, CaMKII, and CREB in hippocampi. Data are expressed as mean ± SEM. ^#^ *p* < 0.05, ^##^ *p* < 0.01 versus the control group; * *p* < 0.05, ** *p* < 0.01 versus the Pilo group.

Western blot assays demonstrated a significant upregulation of NR2B, p-CaMKII, CREB, and p-CREB expression in the hippocampi of mice in the Pilo group (*p* < 0.01). Meanwhile, the protein expression of CaMKII remained unchanged. Administration of Gent (800 mg/kg) resulted in a significant reduction in NR2B, p-CaMKII, CREB, and p-CREB protein expression when compared to the Pilo group (*p* < 0.05) (Figure 6A–F).

To further validate our findings, we examined the impact of Gent on *NR2B*, *CaMKII*, and *CREB* mRNA expression through qPCR analysis. The results demonstrated a significant upregulation of *NR2B*, *CaMKII*, and *CREB* mRNA expression in the Pilo group (*p* < 0.05). The application of Gent at 800 mg/kg resulted in a marked reduction in the mRNA expression of NR2B, CaMKII, and CREB in contrast to the Pilo group (*p* < 0.05) (Figure 6G–I).

#### 2.5.2. Gent Reduced the Expression of Apoptosis-Associated Proteins in the Hippocampus of Lithium/Pilo-Induced Epileptic-Seizure Mice

**Figure 7:**
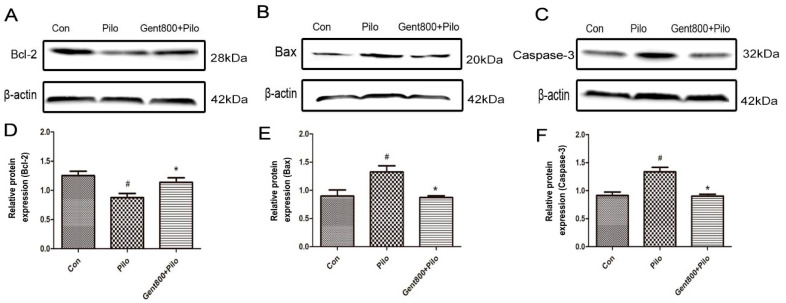
Effect of gentiopicroside on apoptosis-associated protein expression in lithium/Pilo-induced epilepsy seizures mice (n = 6 per group). (**A**–**F**) Western blot and quantitative analysis of Bax, Bcl-2, and Caspase-3 levels. The relative densities are expressed as the mean ± SEM. ^#^ *p* < 0.05 versus the Con group, and * *p* < 0.05 versus the Pilo group.

The levels of apoptotic proteins in hippocampal tissues of mice were measured by performing a Western blot assay, with a particular focus on Bcl-2, Bax, and caspase-3. In the Pilo group, there were significantly decreased levels of the Bcl-2 protein (*p* < 0.05), as well as increased caspase-3 and Bax levels (*p* < 0.05). Administration of Gent (800 mg/kg) prior to lithium/Pilo treatment led to an increase in Bcl-2 protein levels (*p* < 0.05), accompanied by a reduction in both Bax and caspase-3 levels (*p* < 0.05) (Figure 7). These results confirmed that Gent attenuates lithium/Pilo-induced epileptic seizures via decreasing neuron apoptosis in the hippocampus of mice.

### 2.6. Gent Regulating Neuroinflammatory-Related Factors

#### 2.6.1. Gent Diminished Expression of the TLR4/NF-κB Signaling Pathway in the Hippocampus of Lithium/Pilo-Induced Epileptic-Seizure Mice

**Figure 8:**
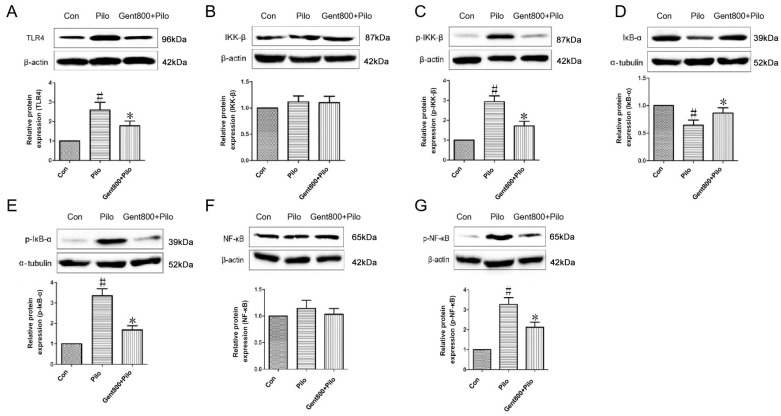
Gentiopicroside diminished the TLR4/NF-κB signaling pathway (n = 6 per group). (**A**–**G**) The level of the TLR4, IKK-β, p-IKK-β, IκB-α, p-IκB-α, NF-κB, and p-NF-κB proteins in the hippocampus were determined by Western blot. Data are expressed as mean ± SEM. ^#^ *p* < 0.05 versus the control group; * *p* < 0.05 versus the Pilo group.

NF-κB is situated in the downstream signaling pathway of TLR4 and is recognized as a critical initiating factor in the inflammatory cascade. As illustrated in Figure 8, our study showed a significant increase in the levels of TLR4, p-IKK-β, p-IκB-α, and p-NF-κB proteins, along with a decrease in the IκB-α protein (*p* < 0.05), while the levels of IKK-β and NF-κB proteins remained unaffected (*p* > 0.05) in the hippocampal tissues of mice after lithium/Pilo administration. Gent administered at 800 mg/kg attenuated the increases in TLR-4, p-IKK-β, p-IκB-α, and p-NF-κB protein levels, and decreases in IκB-α protein levels induced by lithium/Pilo (*p* < 0.05). The protein expression levels of IKK-β and NF-κB remained unaffected (*p* > 0.05). These data suggest Gent ameliorates neuroinflammation in lithium/Pilo-induced epileptic mice through inhibiting the TLR-4-mediated NF-κB signaling pathway.

#### 2.6.2. Gent Reduced the Expression of Pro-Inflammatory Cytokines in the Hippocampus of Lithium/Pilo-Induced Epileptic-Seizure Mice

To ascertain the involvement of neuroinflammation in the therapeutic mechanism of Gent-treated epilepsy seizures in mice, we conducted an evaluation of the protein levels of IL-1β, TNF-α, and IL-6 in hippocampi using Western blot analysis.

**Figure 9:**
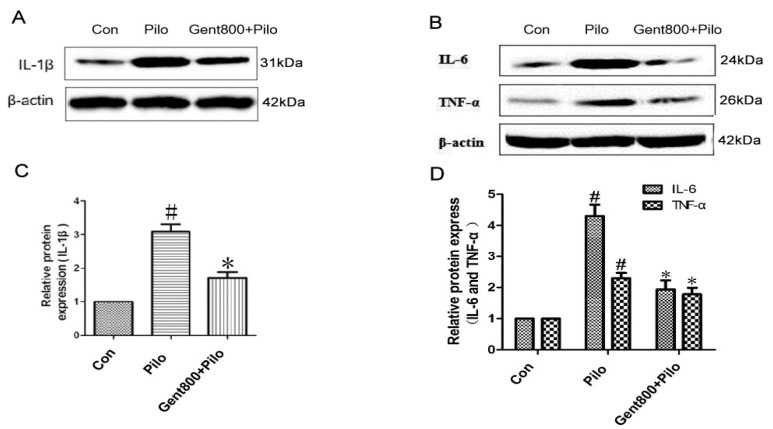
Gentiopicroside reduced the expression of pro-inflammatory cytokines at 24 h after lithium/Pilo-induced epileptic seizures in hippocampus (n = 6 per group). (**A**–**D**) Western blot assay and quantitative analysis of IL-1β, TNF-α, and IL-6. Data are expressed as mean ± SEM. ^#^ *p* < 0.05 versus the control group; * *p* < 0.05 versus the Pilo group.

As shown in Figure 9, our findings revealed a pronounced upregulation of IL-1β, TNF-α, and IL-6 proteins in the hippocampal tissues of mice in the Pilo group compared to the control group (*p* < 0.05). However, treatment with Gent (800 mg/kg) significantly reversed lithium/Pilo-induced elevations in IL-1β, TNF-α, and IL-6 levels (*p* < 0.05). Overall, Gent effectively reduced the expression of pro-inflammatory cytokines in the hippocampi of lithium/Pilo-induced epileptic mice.

#### 2.6.3. Gent Suppressed Lipopolysaccharide (LPS)-Induced Production of Pro-Inflammatory Cytokines in Astrocytes

Literature reports corroborate both in vitro and in vivo experiments indicating that astrocytes are the primary source and target of inflammatory signals induced by epilepsy [43]. Extensive research has confirmed that excessive activation of astrocytes and the subsequent release of various pro-inflammatory cytokines, such as IL-1β, IL-6 and TNF-α, are considerably involved in the pathogenesis and progression of epilepsy.

**Figure 10:**
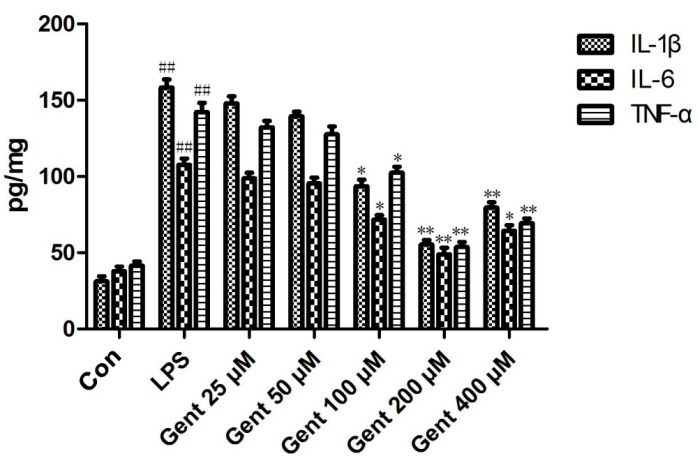
Gentiopicroside reduced LPS-induced pro-inflammatory cytokines expression (n = 6 per group). Data are expressed as mean ± SEM. ^##^ *p* < 0.01 versus the control group; * *p* < 0.05, ** *p* < 0.01 versus the LPS group.

In this experiment, an elevation was noted in the emission of pro-inflammatory mediators, such as IL-1β, IL-6, and TNF-α, by astrocytes in the LPS-induced astrocyte inflammatory response model. Treatment with Gent at concentrations of 100, 200, and 400 μM significantly mitigated the emission of these pro-inflammatory cytokines, indicating that Gent has anti-inflammatory effects on LPS-induced astrocyte inflammation, with the most pronounced effect observed at 200 μM (Figure 10).

#### 2.6.4. The Impact of Gent on Protein Levels Within the TLR-4/NF-κB Signaling Pathway in an LPS-Induced Astrocyte Inflammation Model

**Figure 11:**
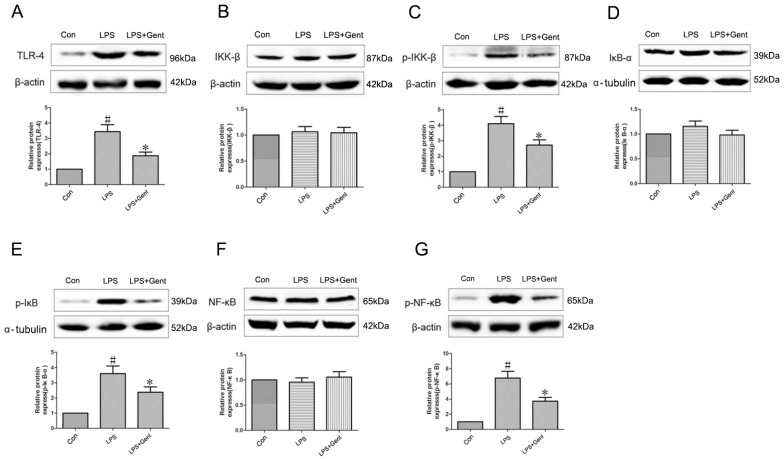
Effect of gentiopicroside on the expression of TLR-4/NF-κB proteins after LPS-induced astrocyte inflammation model (n = 6 per group). (**A**–**G**) The level of the TLR4, IKK-β, p-IKK-β, IκB-α, p-IκB-α, NF-κB, and p-NF-κB proteins in astrocytes were determined by Western blot. Data are expressed as mean ± SEM. ^#^ *p* < 0.05 versus the control group; * *p* < 0.05 versus the LPS group.

The levels of TLR-4, p-IκB-α, p-IKK-β, and p-NF-κB proteins in the LPS-treated group of astrocytes were significantly upregulated (*p* < 0.05). Administration with Gent markedly attenuated the expression of these proteins induced by LPS (*p* < 0.05). Notably, the IKK-α, IKK-β, and NF-κB levels remained unaffected (*p* > 0.05). This confirmed that Gent exerts a suppressive effect on the TLR-4/NF-κB signaling pathway (Figure 11).

## 3. Discussion

Medicinal plants are characterized by abundant resources and their weaker side effects, thus providing a wealth of natural compounds that can be used to develop new herbal remedies. Gent is a secoiridoid substance that has been extracted from *Gentiana macrophylla* Pall., which is an important medicinal plant with a long history of medicinal applications.

This study showed that epileptic seizures in adult mice induced by lithium/Pilo triggered events resulting in aberrant ECoG discharges, generalized seizures, morphological changes associated with neuronal degeneration, and alterations in biochemical markers, including elevated expression of the NR2B/CaMKII/CREB and TLR4/NF-κB signaling pathway proteins. As far as we know, this research is the first to report the possibility of Gent reducing seizure scores, aberrant ECoG discharge, and neuronal degeneration in seizure-prone adult mice. This study indicates that Gent has anticonvulsant effects and neuroprotective properties against seizures caused by lithium/Pilo in a dose-dependent manner. The mechanism of action involves suppressing apoptosis via the NR2B/CaMKII/CREB signaling pathway and suppressing neuroinflammation via the TLR4/NF-κB signaling pathway, hence exerting a significant influence on preservation of neuronal viability. This discovery motivated us to expand the scope of the study to assess the processes that may be responsible for the potential therapeutic benefits of Gent. This study expands the therapeutic applications of Gent; prior research has demonstrated that Gent exhibits therapeutic effects on diabetes, non-alcoholic steatohepatitis, and various other diseases [40,44]. In this study, we have identified its potential therapeutic effects on epilepsy in murine models. Future research will focus on elucidating the specific molecular targets of Gent and modifying its chemical structure to enhance its pharmacological activity and bioavailability.

Seizures and epilepsy must be modeled experimentally to understand the mechanisms underlying ictogenesis and epileptogenesis and to develop therapeutic alternatives [45]. Many epilepsy studies have employed the lithium/Pilo-induced epilepsy model, which uses this agonist of the muscarinic receptor to induce seizures. This model is useful for studying the fundamental mechanisms of epileptogenesis, as it can produce acute seizures, SE, and recurrent spontaneous seizures with great efficacy [46]. In addition to successfully reproducing the behavioral, electrographic, and histological alterations of seizures, the model used in this study also causes early neuronal damage in both mice and rats after lithium/Pilo is administered [47,48].

In this study, administration of Gent notably extended the latency period of seizures and status epilepticus, while concurrently diminishing the severity of seizure activity. The administration of high doses of Gent, specifically at 400 and 800 mg/kg, prolonged the latency to the first convulsion, time to status epilepticus, and reduced mortality in mice. Furthermore, Gent reduced aberrations observed in ECoG measurements, specifically in terms of increased frequency and amplitude, which are associated with alterations in seizure-related behavior. These studies collectively indicate that Gent exerts an anticonvulsant effect on seizures in mice induced by lithium/Pilo.

As a widespread and significant neurological issue, epilepsy is caused by a disruption of the normal balance between inhibitory neurotransmission by GABA and excitatory neurotransmission by glutamate [8]. The distribution of the NMDA glutamate ionotropic receptor subtype is widespread across both the hippocampal and cortical regions. A strong connection exists between the emergence of epilepsy and the activation of NMDA receptors, with evidence from in vivo and in vitro pharmacological and genetic analyses of the receptors [49,50,51]. It is of vital importance that the activation of NMDA receptors facilitates the stimulation of neurons within the central nervous system. The heteromeric NMDA receptor is composed of four distinct subunits. A total of seven isoforms of the NMDA receptor (NR1, NR2A–D, NR3A, and NR3B) have been characterized in previous studies [52]. The NR2B component of the NMDA receptor remains poorly understood in its involvement in epileptic seizures. The available evidence indicates that the expression of NR2B is mostly influenced by behavioral experiences [16,53,54]. The heightened activation of NR2B subunits, induced by the elevated release of extracellular glutamate during seizures, leads to an excessive influx of calcium ions into the neurons. This, in turn, triggers the activation of downstream signaling cascades that are dependent on calcium, involving a range of proteases and phospholipases [55]. The CaMKII factor is recognized for exerting a marked influence on the transfer of calcium information in various neuronal types. This factor can bind calcium and subsequently form complexes with calcium/calcium–calmodulin [56]. The findings of this study indicate that mice treated with lithium/Pilo experience an increase *NR2B* and *CaMKII* mRNA levels, along with an elevation of NR2B and p-CaMKII expression in the hippocampal region. Treatment with Gent (800 mg/kg) prior to lithium/Pilo administration led to a notable decrease in *NR2B* and *CaMKII* mRNA levels and NR2B and p-CaMKII protein expression.

CREB drives gene expression cascades that regulate long-term brain plasticity and neuronal survival [57]. In the management of seizures and epilepsy, the Bcl-2 family of cell death proteins plays a crucial role. The balance between the proapoptotic and antiapoptotic members of this family dictates the course of intrinsic apoptosis. They also control downstream caspase activity [58]. Cytochrome c (Cytc)-releasing reactions can absorb Bax into the mitochondrial membrane. This release activates caspase-3, causing apoptosis, nuclear chromatin condensation, and DNA breaks [51]. The current research identified signs of neurodegeneration in the hippocampal subfields CA1 and CA3 using Nissl and FJB staining in mice administered lithium/Pilo, revealing increased expression of caspase-3 and Bax, and decreased expression of Bcl-2. Treatment with Gent (800 mg/kg) before the Pilo administration reversed these changes, indicating that Gent can improve lithium/Pilo-induced epilepsy by inhibiting apoptosis.

Astrocytes in animal models of epilepsy can be abruptly activated by morphological and functional changes, leading to an increase in GFAP expression. As a cytoskeletal protein specific to astrocytes, GFAP is a reliable marker for identifying the proliferation of reactive astrocytes [59]. The proliferation of reactive astrocytes can lead to excessive excitation of the hippocampal circuit by increasing neuronal glutamine, ultimately resulting in seizures and epilepsy. Activated astrocytes can secrete a significant quantity of pro-inflammatory cytokines. These cytokines are significantly linked with the development of epilepsy [60] and are markedly heightened in epilepsy patients despite their naturally low expression levels in the brain [61]. Also, these cytokines reduce the seizure threshold and alter neuronal excitability, which contribute to an already hyperactive neural network and subsequent spontaneous recurring seizures. Epileptic seizures are caused by IL-1β, a cytokine that promotes inflammation [62,63]. With the activation of astrocytes, the influential pro-inflammatory cytokine IL-1β induces the generation of IL-6 and TNF-α, both of which are strongly linked to epilepsy. Controlling the levels of IL-6 and TNF-α in the brain can lead to a reduction in epileptic events [64]. Therapies for epilepsy now incorporate anti-inflammatory agents that are designed to target specific pro-inflammatory cytokines.

Our findings show that administering Gent (800 mg/kg) notably decreased the number of GFAP-positive cells and the expression of IL-1β, TNF-α, and IL-6 in the hippocampus of lithium/Pilo-induced epileptic mice, while also inhibiting the LPS-induced generation of these pro-inflammatory cytokines in astrocytes. This indicates that Gent inhibits the activation, proliferation, and inflammation of astrocytes induced by lithium/Pilo in epileptic mice.

Toll-like receptors, classified as detectors of patterned molecules, are indispensable in inflammation for recognizing molecular signatures linked to damage [65]. TLR4 is the most extensively and deeply studied subtype and has been shown to be closely related to brain excitability [66]. Significant upregulation of TLR4 expression has also been observed in epilepsy patients and animal models [67,68]. NF-κB, as an important downstream effector of TLR4, is regulated by both IKK and IκB and is an important nuclear transcription factor in regulating immunity and inflammation. Under normal physiological conditions, the intracellular NF-κB inhibitor IκB-α interacts with NF-κB to form a dimer complex that is located in the cytoplasm in an inactive state. When the body’s cells are stimulated by external factors (such as epilepsy), TLR4 is activated, which can then activate IKKs to phosphorylate them. Phosphorylated IKKs can further phosphorylate and activate IκB-α. After phosphorylation, IκB-α is ubiquitinated. NF-κB then dissociates and degrades, activating NF-κB phosphorylation. The phosphorylated NF-κB is then transported to the nucleus, regulating the expression of various pro-inflammatory genes. These inflammatory factors can activate TLR4/NF-κB and related inflammatory signaling pathways on astrocytes and other inflammatory cells, exacerbating the inflammatory response and releasing inflammatory factors, forming a vicious cycle to further promote inflammation. This increases neuronal excitability to contribute to the pathogenesis of epilepsy. The findings of this study indicate that lithium/Pilo decreases the expression of IκB-α, while elevating the expression of TLR4, p-NF-κB, p-IκB-α, and p-IKK-β in the hippocampus. However, there was no change in the protein expression of IKK-β and NF-κB in each group. Administration of Gent (800 mg/kg) prior to therapy resulted in a notable decrease in the expression levels of TLR4, p-NF-κB, p-IκB-α, and p-IKK-β.

## 4. Materials and Methods

### 4.1. Source of Drugs and Reagents

Gentiopicroside was provided by Jingzhu Biotechnology Ltd. (Nanjing, China) with a purity exceeding 98%. Lithium chloride, pilocarpine, and lipopolysaccharide were purchased from Sigma-Aldrich Ltd. (Shanghai, China). Atropine was purchased from Yuanye Biotechnology Co., Ltd. (Shanghai, China).

### 4.2. Animals

The experimental groups comprised adult male ICR mice with an initial weight range of 18–22 g that were obtained from the experimental animal center of Ningxia Medical University (Certificate number: SYXK Ningxia 2015-0001). The mice were accommodated in plastic enclosures, maintained at a controlled temperature ranging from 21 °C to 25 °C, adhering to a 12-h alternating light and dark schedule. The mice had unrestricted access to standardized diet and water. Experimental procedures on animals adhered to the guidelines provided in the National Institutes of Health Guide for the Care and Use of Laboratory Animals and were approved by the Animal Ethics Committee of Ningxia Medical University.

#### Animal Model

Following the weighing and allocation of the animals into cages, a pretreatment was administered via an intraperitoneal injection of lithium chloride at a dosage of 127 mg/kg. After a 24-h interval, an intraperitoneal injection of atropine at a dosage of 1 mg/kg was administered to mitigate the peripheral cholinergic effects induced by Pilo. Subsequently, 30 min post-atropine administration, Pilo was intraperitoneally injected at a dosage of 280 mg/kg to induce an epilepsy model in the mice.

### 4.3. Cell Culture

The MA-c astrocytes obtained from CELLBIO LTD (CBR-131762, Shanghai, China) were maintained in DMEM/F12 (Biological Industries, Kibbutz Beit Haemek, Israel) with 10% fetal bovine serum and 1% penicillin–streptomycin. After 10 to 20 passages, the cells were incubated at 37 °C in a 95% air and 5% CO_2_ atmosphere with humidity maintained at 95%.

To establish an astrocyte model of inflammation, LPS dissolved in normal saline was applied for 24 h at a concentration of 1 μg/mL. Different concentrations of Gent (25 μM, 50 μM, 100 μM, 200 μM, 400 μM) were added 1 h before LPS. The cells were collected for a (3-(4,5-dimethylthiazol-2-yl)-2,5-diphenyltetrazolium bromide) tetrazolium (MTT) assay. Cytocentrifugation was used to collect the supernatant for ELISA and protein was extracted for Western blotting.

After selecting the optimal Gent concentration (200 μM) by ELISA, it was used to treat the astrocytes that were then assessed by Western blotting. The astrocytes were divided into three treatment groups: one serving as the control, another exposed to 1 μg/mL LPS, and the third concurrently treated with 200 μM Gent and 1 μg/mL LPS.

### 4.4. Experimental Group

The mice were randomly assigned into six different experimental groups as follows:

1. Control group: 0.9% normal saline;
2. Pilo group: 0.9% normal saline and Pilo (280 mg/kg);
3. PB 30 mg/kg + Pilo group: phenobarbital dissolved in 0.9% normal saline;
4. Gent 200 mg/kg + Pilo group: Gent dissolved in 0.9% normal saline;
5. Gent 400 mg/kg + Pilo group;
6. Gent 800 mg/kg + Pilo.

Each of the above drugs was administered via intraperitoneal injection. Except for the control group, all groups were given lithium chloride (127 mg/kg), atropine (1 mg/kg), and Pilo (280 mg/kg). We refer to the lithium/pilocarpine group as the “Pilo group” for short.

### 4.5. Behavioural Observation

The evaluation of epileptic seizures was conducted using a modified Racine scale of convulsion grading, a widely recognized and used method for grading convulsions [69]. The grading system was as follows: Class I comprises seizures that display reduced activity levels (hypoactivity) and the occurrence of automatic movements involving the mouth and face. Class II seizures are identified by the presence of repetitive head nodding and an act of chewing or teeth grinding. Class III seizures are identified by the involuntary rhythmic contractions of the muscles in the forelimbs, without rearing. Class IV seizures are classified by the presence of involuntary rhythmic contractions of the muscles in the forelimbs, with an act of rearing. Class V seizures are identified by the occurrence of rearing and the subsequent loss of posture.

Following each lithium/Pilo administration, thorough and uninterrupted monitoring of the mice’s behavior was conducted over 90 min. The purpose of this observation was to identify any obvious patterns of activity exhibited by the mice. The study focused on measuring and observing several key parameters, including the latency to the onset of the first convulsion, specifically class IV–V seizures, and the duration of time until the occurrence of status epilepticus (SE). In addition to evaluating the survival rate within a 24-h timeframe, the duration of SE was also examined. This experiment exposed mice with class IV–V seizures to SE for 90 min. The risk of mortality was reduced by stopping convulsions with a 10 mg/kg intraperitoneal diazepam injection [70].

### 4.6. Cortical Electrocorticography Recording

Each group had eight mice in this experimental design. To induce anesthesia, the mice were subjected to 3.5% chloral hydrate intraperitoneally. Afterward, an inch-long incision was made stereotactically. Monopolar electrodes, composed of stainless steel wire coated in polyurethane of 100 µm diameter, were inserted during surgery. Additionally, electrical resistance was less than 1 Ω/10 mm, suggesting its capacity to block electric current flow over a short distance. The electrode was placed 1.8 mm lateral to the midline and 1.5 mm anterior to the bregma in the left frontal cortex. To accurately record ECoG activity, a reference electrode was implanted in the cerebellum 1.5 mm behind the lambda and directly on the midline [71]. Using dental acrylic, a composite material made of several alloys, the electrodes were attached. After one week, intraperitoneal drugs were given. The ECoG recording was conducted for a duration of 30 min using the SMUP-U4 biological signal processing system, following the administration of the final Pilo injection. The incoming signals underwent a 2000-fold amplification and were subsequently subjected to a bandpass filter with a cut-off frequency ranging from 1 Hz to 50 Hz. During the ECoG recording session, an analysis was conducted to assess both the total power and the mean amplitude [72].

### 4.7. Sample Preparation for Histological Staining

To perform Nissl, Fluoro-Jade B (FJB) and immunohistochemical staining, a group of four animals was included in each experimental group. Deep anesthesia in the mice was induced through an intraperitoneal injection of a solution containing 3.5% chloral hydrate. After a 72-h interval subsequent to the initial occurrence of convulsions or SE, the mice underwent perfusion with a solution consisting of 0.9% saline and 4% paraformaldehyde at a temperature below freezing. The cerebral tissues were extracted and afterwards immersed in a 4% paraformaldehyde solution for a duration of 12 h. Subsequently, the tissues were dehydrated and encased in paraffin. The brains were then cut into 4-micrometer-thick coronal slices. These sections were subjected to dewaxing using xylene and subsequent rehydration using a gradient of ethanol ranging from 100% to 70%. Using a microscope at 400× magnification (Olympus, Tokyo, Japan), the neuronal damage was examined and quantified within the CA1 and CA3 regions.

#### 4.7.1. Nissl Staining

The specimens were subjected to a rinsing procedure using a cresyl violet solution for 1 h at a temperature of 56 °C. Following that, the specimens were promptly immersed in distilled water to remove any residual substances, and then subjected to differentiation in 95% alcohol for 5 min. The slices were later subjected to a dehydration process using alcohol solutions of varying concentrations, with each concentration lasting for 5 min. The slices underwent a clearing process in xylene for 5 min. Finally, they were carefully mounted using a neutral gum solution, as previously published [73]. The remaining pyramidal cells in the hippocampus of CA1 and CA3 regions, with a length of 1 mm each, were quantified in a blinded manner. This quantification was performed on every tenth slice, with a total of six sections analyzed for each animal.

#### 4.7.2. FJB Staining

The application of FJB staining on brain slices has gained significant recognition as a highly reliable technique for identifying neurons in a state of degeneration [71]. The slides were subjected to immersion in a solution containing potassium permanganate at a concentration of 0.06% for 10 min. Later, the slides were subsequently rinsed with distilled water for two minutes. Sections were agitated on a shaker within a mixture comprising 0.1% acetic acid and 0.0004% FJB (Chemicon International, Rolling Meadows, IL, USA). Following a 20-min soak in the staining solution, the slides were subjected to a series of three consecutive rinses in distilled water, with each rinse lasting for one minute. Following the completion of the staining process, the slides were left to air-dry for 5 to 10 min. Before applying a neutral gum solution to the slides, it was necessary to immerse them in xylene for a minimum of one minute. The quantification of neuronal cells positive for FJB staining was conducted by third-party observers who were blinded to the experimental conditions. The assessment was performed in hippocampal CA1 and CA3 regions of 1 mm in length. These regions were examined in every tenth section of the animal, resulting in a total of six sections per animal.

#### 4.7.3. Immunohistochemical Staining

Paraffin sections were heated in a boiling water bath with a 0.01 mol/L sodium citrate solution at pH 6.0 for a duration of 10 min. Once cooled, they were rinsed with PBS for five minutes and the cleaning process was repeated three times. The tissue slices were placed in an antigen retrieval kit containing 0.01 mol/L citrate buffer, boiled in an induction cooker for 10 min, cooled to room temperature, then washed three times with PBS for 5 min each. Goat serum sealing solution was added onto the surface of paraffin tissue sections, which were incubated at room temperature for 10 min to block endogenous peroxides. The sealing solution was shaken off and samples were rinsed with PBS for five minutes, repeating the cleaning process three times. After blocking, the surfaces of brain tissue slices were evenly covered with diluted GFAP primary antibody (1:500) and incubated at 37 °C for two hours. After incubation, they were subjected to three washes with PBS for two minutes each, then incubated with secondary antibody (1:2000) at 37 °C for two hours. After incubation, they were washed three times with PBS for two minutes each. A 3,3’-diaminobenzidine (DAB) working solution was prepared by diluting and mixing 1:50 μL of a concentrated DAB solution with 1 mL of purified water. The DAB working solution was dropped onto the surface of sliced brain tissue and reacted for one minute. Afterward, the tissue was washed three times with PBS for two minutes each. Hematoxylin counterstain was applied for one minute, followed by three washes with tap water for two minutes each. Samples were differentiated with 1% hydrochloric acid and returned to blue with ammonia water. Finally, the film was sealed with neutral gum. The specimens were observed under a microscope after drying. Photos were taken under a 100× eyepiece using a double-blind method to count GFAP-positive cells in the cortex, hippocampal CA1 and CA3 regions, and Hilus region.

### 4.8. qPCR

Total RNA was isolated from the hippocampal tissue samples using TRIzol^®^ (Invitrogen, Carlsbad, CA, USA) following the protocols provided by the manufacturer. RNA concentrations were measured using a quantitative nucleic acid analyzer (Thermo Fisher Scientific, Waltham, MA, USA). Subsequently, the total RNA was converted into complementary DNA (cDNA) using the RevertAid First Strand cDNA Synthesis Kit (Thermo Fisher Scientific, Waltham, MA, USA). The qPCR assay was conducted using the Green qPCR SuperMix provided by Bio-Rad (TransGen, Beijing, China). The relative expression levels of the target mRNA were calculated using the 2^−ΔΔCt^ method, with *β-actin* serving as an endogenous control to normalize the sample data. The primer sequences are shown in Table 2.

### 4.9. ELISA

The concentrations of pro-inflammatory mediators in the astrocyte culture supernatants were quantified using ELISA kits, following the manufacturers’ protocols. Briefly, the standard sample was prepared. The standard sample and the test sample were dispensed into separate wells of a 96-well ELISA plate pre-coated with specific antibodies. A biotinylated antibody specific to the target protein was introduced and incubated for 1 h. Streptavidin–horseradish peroxidase (HRP) was added and incubated for 45 min. Finally, the TMB substrate was added followed by stop solution to induce color development. The absorbance was read at 450 nm using a microplate reader (Thermo Fisher Scientific, Waltham, MA, USA).

### 4.10. Western Blot Assay

The Western blot procedure was executed as previously described. Hippocampal tissues and astrocytes were lysed and the protein concentration of the resulting extracts determined using a bicinchoninic acid (BCA) protein assay kit (Key Gen Biotech, Nanjing, China). A uniform quantity of protein (40 μg) from each experimental group was applied and separated on SDS-PAGE gels, followed by transfer to a nitrocellulose membrane supplied by Millipore, Billerica, MA, USA. Afterward, the membranes were blocked with 5% nonfat milk in TBST at 25 °C for 1 h and incubated with the primary antibodies at 4 °C overnight. The primary antibodies are listed in Table 3. Finally, the membrane was incubated with a horseradish peroxidase (HRP)-conjugated secondary antibody goat anti-rabbit IgG (dilution 1:2000; Protein-Tech, Wuhan, China, catalog number SA00001-2). Positive bands were detected using enzyme-linked chemiluminescence (ECL, APG BIO, Beijing, China). The luminescence of the bands was subsequently analyzed and quantified by densitometry using the Quantity One software (Bio-Rad Laboratories, Hercules, CA, USA).

### 4.11. Statistical Analysis

The statistical analysis of all experimental data was performed using SPSS 18.0 (SPSS Inc., Chicago, IL, USA). The results were presented as the mean (±) SEM. The calculation of the number of animals that exhibited SE and survived was expressed as percentages. These percentages were compared using the χ^2^ test, a nonparametric test. Additional data were compared between more than two groups using the LSD post hoc test after one-way ANOVA, or between two groups using Student’s *t*-test. The statistical significance level was determined at *p* < 0.05.

## 5. Conclusions

The results of this study demonstrate that administering lithium/Pilo caused epileptic seizures in vivo, which, in turn, activated NR2B in hippocampal regions of adult mice. This activation of NR2B may be implicated in the activation of CaMKII and CREB. These findings also highlight the Bcl-2 protein family’s functional role in seizures and hippocampus neuronal deterioration, as well as the part the inflammatory response plays in the progression of epileptic seizures.

At the same time, our study also has some limitations. Firstly, we only validated two signaling pathways but lacked exploration of the targets of Gent. Secondly, the current dosage of Gent is relatively high, and its bioavailability can be improved through structural modifications to make it easier to pass through the blood-brain barrier. Thirdly, it is important to determine the specific region or nucleus of the hippocampus where Gent acts. Fourthly, apart from chemical substances, Gent is also effective in treating epilepsy through other modeling methods. These are our future study directions.

The findings of our study provide evidence that Gent exhibits antiepileptic and neuroprotective properties by suppressing the NR2B/CaMKII/CREB and TLR-4/NF-κB signaling pathways. Based on the aforementioned evidence, it is plausible to consider Gent as a promising therapeutic option for managing epilepsy.

## Figures and Tables

**Table 1:** Effects of varying gentiopicroside doses on the percentage of convulsions, status epilepticus, and survival at 1.5 h post-lithium/Pilo injection in each group.

Groups	Number of Animals/Group	Percentage Convulsion (%)	Percentage SE (%)	Percentage Survival (%)
Con	16	0 (0 of 16)	0 (0 of 16)	100 (16 of 16)
Pilo	16	100 (16 of 16) ^##^	93.7 (15 of 16) ^##^	62.5 (10 of 16)
PB + Pilo	16	0 (0 of 16)	0 (0 of 16)	100 (16 of 16)
Gent200 + Pilo	16	100 (16 of 16)	100 (16 of 16)	87.5 (14 of 16)
Gent400 + Pilo	16	100 (16 of 16)	75.0 (12 of 16)	100 (16 of 16) *
Gent800 + Pilo	16	100 (16 of 16)	56.2 (9 of 16) *	100 (16 of 16) *

Results for percentage seizures, SE, and survival are expressed as percentages. ^##^ *p* < 0.01 versus the Con group; * *p* < 0.05 versus the Pilo group (χ^2^ method and Fischer’s exact probability test).

**Table 2:** Primer sequences.

Gene	Primer	Sequences
*NR2B*	Forward	5′-TGCTGCTCATTGTCTCTGCT -3′
	Reverse	5′-CTTTGCCGATGGTGAAAGAT-3′
*CaMKII*	Forward	5′-TTCAATGCCAGGAGGAAACT-3′
	Reverse	5′-TCACACCATCGCTCTTCTTG-3′
*CREB*	Forward	5′-CTGCCCACTGCTAGTTTGGT-3′
	Reverse	5′-CTGCCCACTGCTAGTTTGGT-3′
*β-actin*	Forward	5′-GAGACCTTCAACACCCCAGC-3′
	Reverse	5′-ATGTCACGCACGATTTCCCC-3′

**Table 3:** List of primary antibodies.

Antibody Type	Antibody Target	Dilution	Company	Country	Catalog Number
Rabbit	Bax	1:2000	Protein-Tech	China	50599-2-Ig
	Bcl-2				26593-1-AP
	TLR4				19811-1-AP
	NF-κB p65		Abcam	USA	Ab32536
	IL-1β	1:1500	Protein-Tech	China	26048-1-AP
	NR2B	1:1000	Abcam	USA	Ab254356
	CaMKII				Ab134041
	CREB				Ab32515
	p-NF-κB p65				Ab239882
	Caspase-3				Ab32351
	IKK-β		SAB	USA	#24063
	IKB-α				#13376
	IL-6		Protein-Tech	USA	26404-1-AP
	p-CaMKII (Thr 286)	1:500	Abcam	USA	Ab171095
	p-CREB (Ser 133)				Ab32096
	TNF-α				Ab183218
	p-IKK-β (Tyr188)		SAB	USA	#11929
	p-IKB-α (Ser32/Ser36)				#11152
	β-actin	1:2000	Protein-Tech	China	20536-1-AP
	α-tubulin	1:20,000	Protein-Tech	China	11224-1-AP

## Data Availability

The data presented in this study are available on request from the corresponding authors.
